# Effects of UHDR and Conventional Irradiation on Behavioral and Cognitive Performance and the Percentage of Ly6G+ CD45+ Cells in the Hippocampus

**DOI:** 10.3390/ijms241512497

**Published:** 2023-08-06

**Authors:** Ariel Chaklai, Pamela Canaday, Abigail O’Niel, Francis A. Cucinotta, Austin Sloop, David Gladstone, Brian Pogue, Rongxiao Zhang, Jacob Sunnerberg, Alireza Kheirollah, Charles R. Thomas, P. Jack Hoopes, Jacob Raber

**Affiliations:** 1 Department of Behavioral Neuroscience, Oregon Health Science University, Portland, OR 97239, USA; chaklai@ohsu.edu (A.C.); oniela@ohsu.edu (A.O.); 2 Knight Flow Cytometry Core OHSU, Portland, OR 97239, USA; canadayp@ohsu.edu; 3 Department of Health Physics and Diagnostic Sciences, University of Nevada, Las Vegas, NV 89154, USA; francis.cucinotta@unlv.edu; 4 Department of Radiation Oncology, Geisel School of Medicine, The Thayer School of Engineering, The Dartmouth Cancer Center, at Dartmouth College and the Dartmouth-Hitchcock Medical Center (DHMC), Hanover, NH 03755, USA; austin.m.sloop.th@dartmouth.edu (A.S.); david.j.gladstone@dartmouth.edu (D.G.); jacob.p.sunnerberg.th@dartmouth.edu (J.S.); alireza.kheirollah@dartmouth.edu (A.K.); p.jack.hoopes@dartmouth.edu (P.J.H.); 5 Department of Medical Physics, University of Wisconsin, Madison, WI 53705, USA; bpogue@wisc.edu; 6 Department of Radiation Medicine, New York Medical College, Westchester Medical Center, Valhalla, NY 10595, USA; rongxiao.zhang@wmchealth.org; 7 Departments of Neurology and Radiation Medicine, Division of Neuroscience ONPRC, Oregon Health & Science University, Portland, OR 97239, USA

**Keywords:** mouse, radiation, object recognition, home cage activity, flow cytometry

## Abstract

We assessed the effects of conventional and ultra-high dose rate (UHDR) electron irradiation on behavioral and cognitive performance one month following exposure and assessed whether these effects were associated with alterations in the number of immune cells in the hippocampus using flow cytometry. Two-month-old female and male C57BL/6J mice received whole-brain conventional or UHDR irradiation. UHDR mice were irradiated with 9 MeV electrons, delivered by the Linac-based/modified beam control. The mice were irradiated or sham-irradiated at Dartmouth, the following week shipped to OHSU, and behaviorally and cognitively tested between 27 and 41 days after exposure. Conventional- and UHDR-irradiated mice showed impaired novel object recognition. During fear learning, conventional- and UHDR-irradiated mice moved less during the inter-stimulus interval (ISI) and UHDR-irradiated mice also moved less during the baseline period (prior to the first tone). In irradiated mice, reduced activity levels were also seen in the home cage: conventional- and UHDR-irradiated mice moved less during the light period and UHDR-irradiated mice moved less during the dark period. Following behavioral and cognitive testing, infiltrating immune cells in the hippocampus were analyzed by flow cytometry. The percentage of Ly6G+ CD45+ cells in the hippocampus was lower in conventional- and UHDR-irradiated than sham-irradiated mice, suggesting that neutrophils might be particularly sensitive to radiation. The percentage of Ly6G+ CD45+ cells in the hippocampus was positively correlated with the time spent exploring the novel object in the object recognition test. Under the experimental conditions used, cognitive injury was comparable in conventional and UHDR mice. However, the percentage of CD45+ CD11b+ Ly6+ and CD45+ CD11b+ Ly6G- cells in the hippocampus cells in the hippocampus was altered in conventional- but not UHDR-irradiated mice and the reduced percentage of Ly6G+ CD45+ cells in the hippocampus might mediate some of the detrimental radiation-induced cognitive effects.

## 1. Introduction

After the cessation of whole-brain radiotherapy with X-rays or γ-rays for brain tumors, over 50% of cancer survivors report impaired cognition [1]. Many studies, including those involving 18–24 Gy or 36 Gy of irradiation in children and 60 Gy in adults, with a dose rate between 0.01 and 0.083 Gy/s, have reported the nature and extent of various impairments that are inherently difficult to quantify, and often underestimate the severity of patient complaints that are detected in the clinic [2,3,4,5]. Treatment-associated effects on mood, memory, concentration, and executive functions reported are persistent and have a major negative impact on quality of life. The use of more recent treatment modalities, such as ultra-high dose rate (UHDR) irradiation, with a dose rate larger than 40 Gy/s, increases the need for research with preclinical models to quantify these risks to patients. 

Following promising results in a mouse model of gastrointestinal (GI) injury using high dose rates in the 1960s and 1970s [6,7], a reemergence of interest in ultra-high dose rate (UHDR) therapy has occurred in the last few years due to experiments showing reduced skin, GI, and brain tissue and normal tissue damage with similar tumor control as found with conventional irradiation [8,9,10,11,12,13] and has been called the UHDR effect. This led to the development of a few facilities using electron, X-ray, or proton irradiation to study UHDR irradiation in mouse models [14], including the Dartmouth facility using 10 MeV electrons with dose rates as high as 300 Gy/s used in the current study [15]. Some studies show reduced cognitive injury using UHDR irradiation. Montay-Gruel et al. [11] used whole-brain irradiation (WBI) of female C57BL/6J (age 2 months) mice with a dose of 10 Gy of 4.5 MeV electrons, while varying the dose rate from 0.1 to 500 Gy/s, to show that novel object recognition (NOR) was not affected for dose rates >60 Gy/s. A study involving the same mouse model [16] using synchrotron X-rays with an average dose rate of 37 Gy/s showed no NOR detriments for UHDR irradiation which do occur for conventional X-rays at 2 and 6 months post-irradiation (IR). In addition, UHDR irradiation reduced hippocampal cell-division impairment and induced less reactive astrogliosis [17] compared to conventional IR. A study involving female C57BL/6J mice (age 2 months) [9], using WBI with 6 MeV electrons (dose rate > 100 Gy/s), showed reduced impacts on measures of anxiety and depression and on performance in cognitive tests related to extinction memory at 10 or 12 Gy, but similar measures at 14 Gy compared to conventional IR. A similar study [12] involved 3-week-old C57BL/6J mice undergoing whole WBI with 8 Gy of UHDR (6 MeV electrons with a single pulse at 4.4 × 10^6^ Gy/s) or conventional IR. Simmons et al. [10] used 3-month-old male C57BL/6J mice and WBI at 30 Gy with 16 (200 Gy/s) or 20 MeV (300 Gy/s) electrons. At 10 weeks post-IR, UHDR irradiation did not impair NOR or novel location recognition, and reduced hippocampal dendritic spine loss and neuroinflammation occurred compared to conventional IR. While these studies show the benefits of UDHR irradiation, they are limited by the scope of the cognitive tests, with regard to comparisons of age, sex and dose and dose fractionation, and time at assessment. Only one study involving mouse models of glioblastoma showed a UHDR sparring effect in female mice relying strictly on the novel object recognition test [18]. Therefore, to determine whether UHDR has protective effects on behavioral and cognitive performance, in the current study we assessed the effects of conventional and UHDR irradiation on behavioral and cognitive performance one month following exposure. In addition, we used flow cytometry to assess whether the effects of conventional and UHDR irradiation were associated with alterations in the number of immune cells in the hippocampus and, if so, if the effects of UHDR were associated with fewer alterations in the number of immune cells in the hippocampus than those of conventional irradiation.

## 2. Results

**Figure 1:**
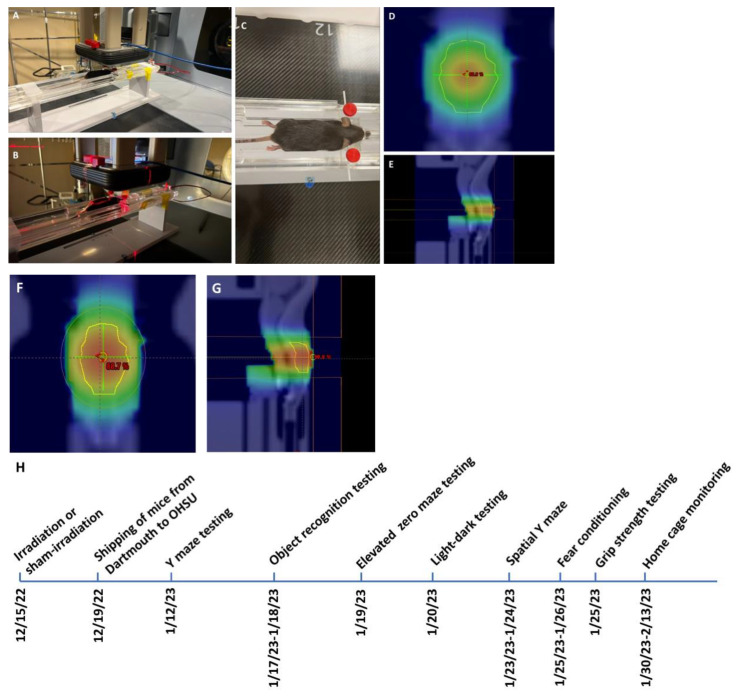
(**A**–**C**) Images of the radiation setup. (**D**,**E**) Two dose wash maps for the UHDR normalized to 100%. D = 5 mm. The sagittal view is at the midplane of the brain. The AP is 5 mm below the top of the mouse. (**F**,**G**) TPS of conventional beamline when calculated at 9 MeV. These are similarly aligned and normalized. (**H**) Timeline of behavioral testing.

*Irradiation and timeline of behavioral testing.* Mice received whole brain conventional or UHDR irradiation as indicated in Table 1 and Figure 1. 

*Y maze.* Irradiation did not affect spontaneous alternation or entries, an activity measure, in the Y maze (Supplementary Figure S1). 

**Figure 2:**
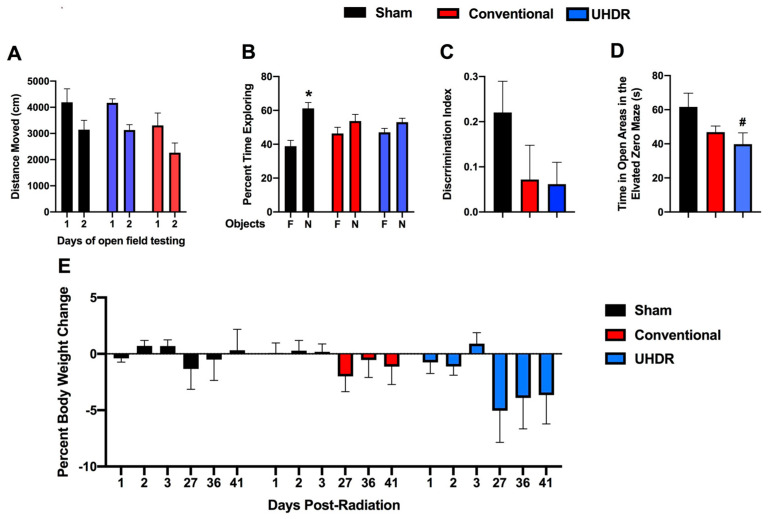
(**A**) Irradiation did not affect activity levels in the open field containing objects. The data for the two days of open-field testing are shown. (**B**) Sham-irradiated mice spent more time exploring the novel than familiar objects; conventional- and UHDR-irradiated mice did not. F: familiar; N: novel. * *p* < 0.05, paired *t*-test. (**C**) While only sham-irradiated mice had a positive discrimination index different from 0, consistent with a preference for exploring the novel object, there was no significant difference in discrimination index between the three groups. (**D**) There was a trend toward an effect of radiation on measures of anxiety in the elevated zero maze with higher measures of anxiety in UHDR- than in sham-irradiated mice. ^#^ *p* = 0.0592, Dunnett’s. (**E**) Irradiation did not significantly affect the percentage of body weight change in the mice.

*Novel object recognition.* In the open field containing the objects, all groups moved less on day 2 than on day 1 (F(1, 14) = 37.85, *p* < 0.001) but there was no effect of treatment on activity levels over the two days of testing (Figure 2A). We also calculated the difference moved over the two days in individual mice and there was no effect of treatment on this performance measure either (sham: 1045 ± 237; conventional: 1039 ± 223; UHDR: 1041 ± 424). 

Sham-irradiated mice showed novel object recognition and spent more time exploring the novel than the familiar object (t = 3.185, *p* = 0.0244, paired *t*-test). However, conventional- (t = 0.9447, *p* =0.3882) and UHDR-irradiated (t = 1.266, *p* = 0.2743) mice did not (Figure 2B). When we analyzed the discrimination index, sham-irradiated mice had a positive discrimination index difference from 0 (t = 3.183, *p* = 0.0097), while conventional- (t = 0.9447, *p* = 0.3671) and UDHR-irradiated mice did not (t = 1.266, *p* = 0.2413) (Figure 2C). However, there was no significant difference in the discrimination index between the three experimental groups.

*Elevated zero maze.* Next, measures of anxiety were assessed in the elevated zero maze. There was a trend toward an effect of irradiation on measures of anxiety in the elevated zero maze (F(2, 14) = 1.255, *p* = 0.0820) and a trend toward higher measures of anxiety in UHDR- than sham-irradiated mice (*p* = 0.0592, Dunnett’s) (Figure 2D).

*Body weights.* There was no effect of irradiation on the percentage of body weight change (calculated based on the pre-exposure body weight) in the mice (Figure 2E). Visual inspection suggests that there might be body weight loss in UHDR-irradiated mice starting at 27 days post-exposure.

*Light–dark test.* In the light–dark test, the mice spent more time in the dark than in the light compartment (F(1, 14) = 65.02, *p* < 0.0001) but there was no effect of radiation on the percentage of time spent in the light or dark compartment (Supplementary Figure S2A) or the number of entries into the more anxiety-provoking light compartment (Supplementary Figure S2B).

*Spatial Y maze.* There was a trend toward an effect of radiation on the percentage of entries into the novel arm of the spatial Y maze (F(2, 14) = 2.947, *p* = 0.0885), with a trend toward a higher percentage of entries in UHDR- than sham-irradiated mice (*p* = 0.0830) (Supplementary Figure S3A). There was no effect of radiation on the percentage of time spent in the novel arm (Supplementary Figure S3B). When the number of arm entries was analyzed, there was a radiation × arm interaction (F(4, 28) = 2.812, *p* = 0.0443) (Supplementary Figure S3C), with a trend toward less arm 2 entries in UHDR- than sham-irradiated mice (*p* = 0.08, Dunnett’s). 

**Figure 3:**
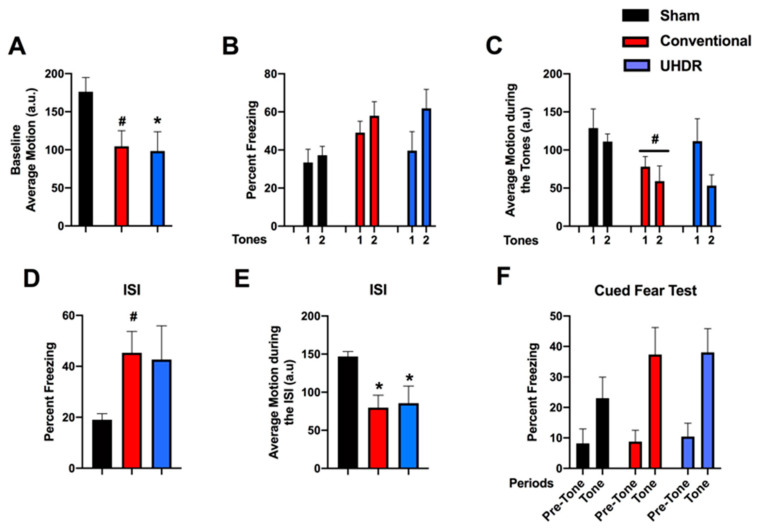
(**A**) During the baseline period of fear learning (prior to the first tone), UHDR-irradiated mice moved less than sham-irradiated mice. * *p* = 0.0436, Dunnett’s. There was also a trend toward conventional-irradiated mice moving less than sham-irradiated mice. ^#^ *p* = 0.0514, Dunnett’s). (**B**) There was no effect of radiation on the percentage freezing during the tones. (**C**) When activity levels during the tones were analyzed, there was a trend toward lower activity levels in conventional-irradiated than sham-irradiated mice. ^#^ *p* = 0.09). (**D**) There was a trend toward higher freezing levels in conventional- than sham-irradiated mice. ^#^ *p* = 0.0757, Dunnett’s. (**E**) There was an effect of radiation on activity levels during the ISI (F(2, 14) = 5.861, *p* = 0.0142) with lower activity levels in conventional-irradiated and UHDR-irradiated than sham-irradiated mice. * *p* < 0.05, Dunnett’s. (**F**) In the cued fear memory test, there was an effect of the period on freezing (F(1, 14) = 54.62, *p* < 0.0001) with higher freezing levels during the tone than during the pre-tone period but there was no effect of radiation on cued fear memory.

*Fear learning and cued fear memory.* During the baseline period of fear learning (prior to the first tone), there was an effect of radiation on activity levels (F(2, 14) = 4.200, *p* = 0.0372) (Figure 3A). UHDR-irradiated mice moved less than sham-irradiated mice (*p* = 0.0436, Dunnett’s) and there was a trend toward conventional-irradiated mice moving less than sham-irradiated mice (*p* = 0.0514, Dunnett’s). There was no effect of radiation on the percentage of freezing during the tones (Figure 3B). When activity levels during the tones were analyzed, there was an effect of tone (F(1, 14) = 8.385, *p* = 0.0117) and a trend toward lower activity levels in conventional-irradiated than sham-irradiated mice (*p* = 0.09) (Figure 3C). Performance during the inter-stimulus interval (ISI) is considered the best measure of fear learning in the fear conditioning test. There was a trend toward an effect of irradiation on freezing levels during the ISI (F(2, 14) = 2.967, *p* = 0.0843) and a trend toward higher freezing levels in conventional- than sham-irradiated mice (*p* = 0.0757, Dunnett’s) (Figure 3D). There was an effect of radiation on activity levels during the ISI (F(2, 14) = 5.861, *p* = 0.0142) with lower activity levels in conventional-irradiated (*p* = 0.0139, Dunnett’s) and UHDR-irradiated (*p* = 0.0307) than sham-irradiated mice (Figure 3E). On day 2, cued fear memory was assessed by placing mice in a new environment. The mice were habituated to the new environment for 90 s (pre-tone), and exposed to the tone (cue) for 180 s. There was an effect of the period on freezing (F(1, 14) = 54.62, *p* < 0.0001), with higher freezing levels during the tone than during the pre-tone period (Figure 3F). There was also an effect of the period on activity levels (F(1, 14) = 19.56, *p* = 0.0006), with lower activity levels during the tone than pre-tone but there was no effect of radiation on cued fear memory.

**Figure 4:**
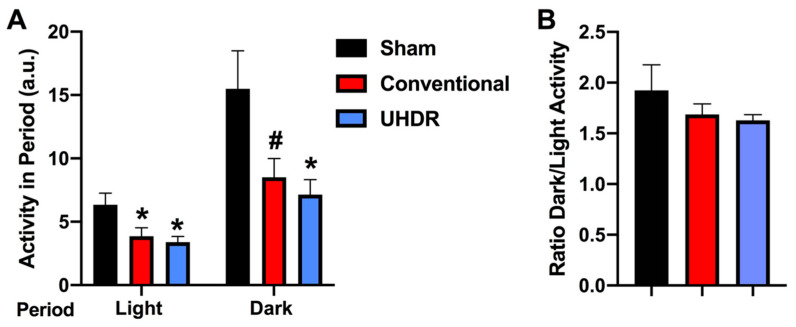
(**A**) During the light period, there was an effect of radiation (F(2, 14) = 4.804, *p* = 0.0258) with lower activity levels in conventional- and UHDR-irradiated mice than sham-irradiated mice. There was also an effect of radiation during the active dark period (F(2, 14) = 4.418, *p* = 0.0258), with lower activity levels in UHDR- than sham-irradiated mice and a trend toward lower activity levels in conventional- than UHDR-irradiated mice. * *p* < 0.05, Dunnett’s, ^#^ *p* =0.0589, Dunnett’s. (**B**) There was no effect of radiation on the ratio of dark/light activity.

*Circadian home cage activity.* Circadian activity levels were assessed over two weeks using infrared sensors in individually housed mice. During the light period, there was an effect of radiation (F(2, 14) = 4.804, *p* = 0.0258) with lower activity levels in conventional- (*p* =0.0475, Dunnett’s) and UHDR-irradiated (*p* =0.0256, Dunnett’s) mice than sham-irradiated mice (Figure 4A). There was also an effect of radiation during the active dark period (F(2, 14) = 4.418, *p* = 0.0258), with lower activity levels in UHDR- than sham-irradiated mice (*p* = 0.0315, Dunnett’s) and a trend toward lower activity levels in conventional- than UHDR-irradiated mice (*p* =0.0589, Dunnett’s) (Figure 4A). Consistent with this pattern, there was no effect of radiation on the ratio of dark/light activity (Figure 4B).

**Figure 5:**
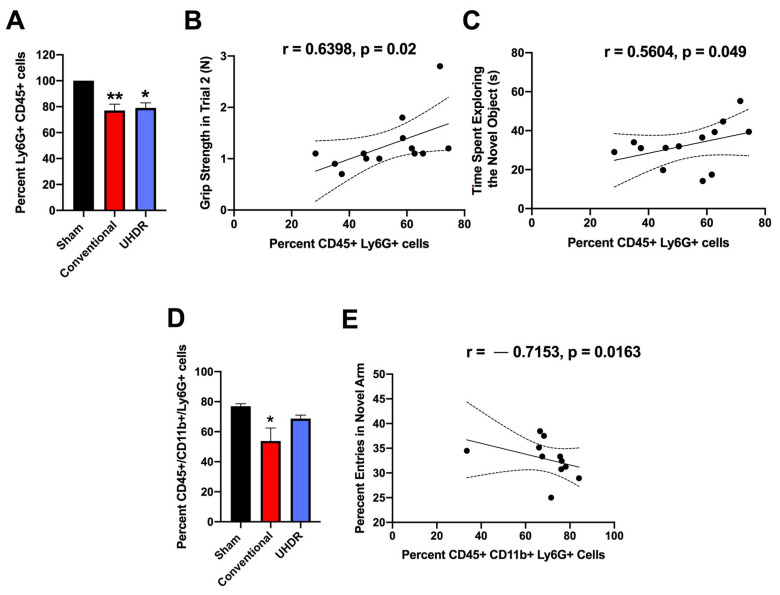
(**A**) There was an effect of radiation on the percentage of Ly6G+ CD45+ cells in the hippocampus (F(2, 12) = 10.82, *p* = 0.0005, Kruskall–Wallis). The percentage of Ly6G+ CD45+ cells in the hippocampus was lower in conventional- and UHDR-irradiated than in sham-irradiated mice. ** *p* < 0.01, * *p* < 0.05. (**B**) The percentage of Ly6G+ CD45+ cells in the hippocampus correlated positively with performance in the second trial of the grip strength test (r = 0.6398, *p* = 0.02, 2-tailed Spearman). Best-fit linear regressions with 95% confidence intervals are shown. (**C**) The percentage of Ly6G+ CD45+ cells in the hippocampus correlated positively with the time spent exploring the novel object in the object recognition test (r = 0.5604, *p* = 0.049, 2-tailed Spearman). (**D**) The percentage of CD45+ CD11b+ Ly6G+ cells in the hippocampus was lower in conventional than sham-irradiated mice. * *p* < 0.05 versus sham-irradiation. (**E**) The percentage of CD45+ CD11b+ Ly6G+ cells in the hippocampus correlated negatively with the percentage of entries in the novel arm of the spatial Y maze. r = −0.7153, *p* = 0.0163, Spearman.

*Flow cytometry.* There was an effect of radiation on the percentage of CD45+ Ly6G+ cells in the hippocampus (F(2, 12) = 10.82, *p* = 0.0005, Kruskall–Wallis) with a lower percentage in conventional- (*p* = 0.0066, Dunn’s) and UHDR-irradiated (*p* = 0.0190, Dunn’s) than sham-irradiated (Figure 5A) mice. As there were three samples for sham- and conventional-irradiated mice, we also analyzed these data without normalization. When sham- and conventional-irradiated female samples were analyzed without normalization, the percentage of Ly6G+ CD45+ cells in the hippocampus was also lower in conventional- (33.6 ± 2.7%) than in sham-irradiated mice (47.1 ± 1.7%) (t = 4.327, *p* = 0.0133, two-tailed *t*-test). There was no effect of radiation on the percentage of CD11b+ CD45+ cells, CD45+ cells expressing high levels of CD45, CD45+ cells expressing low levels of CD45, or 206+ CD45+ cells.

The percentage of CD45+ Ly6G+ cells in the hippocampus correlated positively with performance in the second trial of the grip strength test (r = 0.6398, *p* = 0.02, two-tailed Spearman, Figure 5B). The percentage of CD45+ Ly6G+ cells in the hippocampus also correlated positively with the time spent exploring the novel object in the object recognition test (r = 0.5604, *p* = 0.049, two-tailed Spearman, Figure 5C). 

We next analyzed the effects of irradiation on the percentage of CD45+ CD11b+ Ly6G+ and CD45+ CD11b+ Ly6G- cells in the hippocampus. There was an effect of irradiation on the percentage of CD45+ CD11b+ Ly6G+ in the hippocampus (F(2, 12) = 5.348, *p* = 0.0218, ANOVA), with a lower percentage of CD45+ CD11b+ Ly6G+ cells in conventional than sham-irradiated mice (*p* = 0.0130, Figure 5D). The percentage of CD45+ CD11b+ Ly6G+ cells in the hippocampus correlated negatively with the percentage of entries in the novel arm of the spatial Y maze (r = -0.7153, *p* = 0.0163, Spearman, Figure 5E). 

## 3. Discussion

In the current study, conventional- and UHDR-irradiated mice showed impaired novel object recognition. Consistent with these findings, in the only study that involved NOR testing at one-month post-exposure, comparable to the timeline of our study, NOR was impaired in both conventional- and UHDR-irradiated female nude (NU_(Ico)_-Foxn1^nu^) mice at 14 Gy, a dose similar to the 16 Gy in our study. Preservation of NOR in UHDR- but not conventional-irradiated mice was seen at four months, but not two months, following exposure to 10 Gy [12]. These data indicate that the effects of UHDR might be dose- and post-exposure-interval-dependent. During fear learning, conventional- and UHDR-irradiated mice moved less during the ISI, and UHDR-irradiated mice also moved less during the baseline period (prior to the first tone). In irradiated mice, reduced activity levels were also seen in the home cage: conventional- and UHDR-irradiated mice moved less during the light period and UHDR-irradiated mice moved less during the dark period. Ly6G is a marker of neutrophils. The percentage of Ly6G+ CD45+ cells in the hippocampus was lower in conventional- and UHDR-irradiated than sham-irradiated mice, suggesting that neutrophils might be particularly sensitive to radiation. As the Ly6G+ CD45+ cells in the hippocampus were positively correlated with the time spent exploring the novel object in the object recognition test, the reduced percentage of Ly6G+ CD45+ cells in the hippocampus might mediate some of the detrimental radiation-induced cognitive effects. The percentage of CD45+ CD11b+ Ly6G+ in the hippocampus was lower in conventional than sham-irradiated mice and correlated negatively with the percentage of entries in the novel arm of the spatial Y maze.

In the context of cancer involving (brain) tumors, reduced neutrophil levels are likely also beneficial for tumor control, as neutrophils were shown to promote tumor resistance to radiotherapy, while a lower neutrophil count following chemoradiotherapy was associated with higher rates of local tumor control, metastasis-free survival, and overall survival [19,20,21]. Consistent with these data, an increased neutrophil-to-lymphocyte ratio following neoadjuvant radiotherapy is associated with poor survival outcomes in patients with rectal cancer [22]. In addition, in a mouse model of pancreatic ductal adenocarcinoma in which anorexia and muscle catabolism are seen, brain infiltrating immune cells were mostly neutrophils that expressed the chemokine receptor C-C chemokine receptor type 2 (CCR2) and blocking CCR2 decreased the brain infiltration of the immune cells and cachexia [23]. CCR2 deficiency also protected cognitive function and hippocampal neurogenesis in mice receiving traumatic brain injury, irradiation, or receiving both [24]. The age at irradiation and the activational state of the neutrophils might be important to consider. Neutrophil-specific deletion of a kinase critical in the activational state of neutrophils improved cognitive function following traumatic injury to the developing brain [25]. Conventional, but not UHDR, irradiation reduced the percentage of CD45+ CD11b+ Ly6G+ in the hippocampus. This differential response and more desired response after UHDR than conventional irradiation might be beneficial for tumor control, as CD11+ Ly6G+ cells were shown to inhibit tumor growth [26]. However, this conventional radiation effect might be beneficial for cognitive function, as a lower percentage of CD45+ CD11b+ Ly6G+ in the hippocampus was associated with enhanced cognitive performance in the spatial Y maze.

Recent studies of UHDR irradiation on cognitive performance have used a variety of irradiation delivery systems. They include electron [8,11,27], X-ray [13], and proton irradiation [10]. Electron irradiation in previous studies has been carried out at energies of 4.5 and 6 MeV [8,27] or 16 and 20 MeV [9], while the current study used 9 MeV electrons. Other important beam parameters include the instantaneous dose rate, number of pulses, and pulse width. Our study used an ultra-high instantaneous dose rate (>1000 Gy/s) with a 360 Hz repetition rate of 3 × 10^−6^ s per pulse, which is similar to several past studies with electrons [8,9,27]; however, this will be improved in the future studies at Dartmouth through the implementation of a high-intensity single pulse system. The dose chosen for this study is relevant to tissue irradiation near a tumor volume in cancer therapy and similar to recent studies allowing for comparison of observations found. In the future, multiple-dose and fractionated-dose studies will be considered. 

In the current study, there was a trend toward increased measures of anxiety in the elevated zero maze. Consistent with this trend, when activity levels in the center of the open field were assessed by beam brakes in neonatally irradiated rats, they were lower in conventional- and UHDR-irradiated rats that received 8 Gy and conventional-irradiated rats that received 5 Gy and they seemed lower in 5 Gy UHDR-irradiated rats as well, although it did not reach significance [28]. These data suggest the importance of including measures of anxiety in these brain radiation studies. There was no effect of irradiation on the percentage of body weight change in the mice but visual inspection suggested that there might be body weight loss in UHDR-irradiated mice starting at 27 days post-exposure. Consistent with this pattern, in neonatal irradiated rats, there was no change in body weight up to 24 days after exposure, but starting at 31 days following exposure body weights were lower in conventional- and UHDR-irradiated rats that received 8 Gy [28]. There was no effect of radiation on cued fear memory, consistent with the lack of an effect of conventional and UHDR irradiation in neonatal rats exposed to 5 or 8 Gy [11].

We did not see reduced cognitive injury following UHDR, as compared to conventional cranial irradiation. Together with other studies, these data suggest that the protective cognitive effects of UHDR irradiation might be very dose-dependent and seen at lower and higher doses than those used in the current study. In addition, there is likely a dependence on cognitive tests used to compare conventional to UHDR irradiation. Protective effects of UHDR irradiation for novel object recognition memory were seen at doses of 8, 10, or 12 Gy in female mice but not when the dose was 14 Gy [5,12,29]. Protective effects in the novel object recognition test were also seen in male mice receiving 30 Gy of UHDR irradiation [9]. Together, these data suggest that the protective effects of UHDR on cognitive performance might involve a bell-shaped curve but additional studies involving mice of both sexes and involving a battery of behavioral measures would be required to fully model the response to UHDR and conventional irradiation.

Infiltration of immune cells into the brain might play a role in cognitive injury. For example, in mice with tauopathy and in Alzheimer’s disease (AD) brains, there is microglia-mediated T-cell infiltration-induced tauopathy, a marker of AD neuropathology [30]. The number of T cells, especially cytotoxic T cells, was increased in areas with tau pathology and correlated with neuronal loss, and the cells dynamically transformed their cellular characteristics from activated to exhausted states. When tauopathy mice were given a drug or an antibody known to result in the death of microglia or T cells, both decreased brain atrophy. Depletion of microglia reduced the number of T cells in the brain and T-cell depletion reverted microglia to a state more like that seen in a healthy brain. 

While cognitive injury was comparable in conventional and UHDR mice, the percentage of CD45+ CD11b+ Ly6+ cells in the hippocampus cells in the hippocampus was altered in conventional- but not UHDR-irradiated mice. As the percentage of CD45+ CD11b+ Ly6+ cells correlated negatively with the percentage of entries in the novel arm of the spatial Y maze, a cognitive measure not affected by conventional irradiation, and CD11+ Ly6G+ cells were shown to inhibit tumor growth [26], these data suggest that some immune measures in the hippocampus might have opposite effects on cognition versus tumor control. Future efforts are warranted to increase our understanding of the role of immune cells infiltrating the brain in the detrimental effects of cranial irradiation on cognition in the absence and presence of tumors.

## 4. Materials and Methods

*Mice and irradiation.* Two-month-old female and male C57BL/6J mice (*n* = 9 mice/sex, or 18 mice in total) purchased from JAX labs received whole brain conventional or UHDR irradiation. In order to not limit the response to whole brain conventional or UHDR irradiation to one sex, both sexes were included in the current study. One UHDR-irradiated female mouse died, so there were five mice in that group. UHDR dose mice were irradiated with 9 MeV electrons with a target dose of 18 Gy delivered by the Linac-based/modified beam control. The delivered UHDR dose was verified by GAF chromatic film after irradiation. The conventional dose rate was delivered to match the UHDR. Twenty-four hours is typically allowed between UHDR and conventional irradiations to allow the film to stabilize, however, scheduling constraints prevented this stabilization time between irradiations (in these mice). Early estimates from the film (within 1 h of irradiation) indicated a delivered dose of 18 Gy UHDR and so this dose was delivered to the conventional-irradiated group. Conventional mice were given 18 Gy (MUs calculated from film under a similar setup) to match the UHDR mice. After 24 h passed, the films were reanalyzed and it was found that the early estimates were high, only 17 Gy was delivered in the UHDR group (see Table 1 for details). There were six mice per treatment group, with three mice per sex per group. The mice were irradiated or sham-irradiated at Dartmouth and the following week shipped to OHSU. Following three weeks of quarantine, the mice were behaviorally and cognitively tested between 27 and 41 days after exposure, as described below (see Figure 1F). Subsequently, the mice were tested for circadian home cage activity over two weeks. Mice were group housed at OHSU on a ventilated Thoren rack, except when mice were singly housed for monitoring of home cage activity using a conventional Metro rack and the MLog (BioBServe, Bonn, Germany) home cage sensor system following the week following fear conditioning, as described in [27].

All mice were kept under a constant 12 h light:12 h dark cycle, and water and food (PicoLab Rodent Diet 20, no. 5053; PMI Nutrition International, St. Louis, MO, USA) were provided ad libitum. All procedures were approved by the Institutional Animal Care and Use Committees at Dartmouth and OHSU and were in compliance with all federal regulations. Ten weeks following radiation or sham irradiation, the mice were euthanized by cervical dislocation, and their hippocampi were analyzed for alterations in the numbers of immune cells by flow cytometry.

*Spontaneous alternation in the Y maze.* Spontaneous alterations were assessed in a Y-shaped maze with raised sides (3.8 cm bottom width, 12.55 cm, top width, 12.55 cm height) and made of non-reflective opaque gray plastic (37.98 cm length) (O’Hara & Co., Ltd., Tokyo, Japan) as described in [31]. At the beginning of each 5 min trial, mice were placed into the center of the maze. To isolate the mice from the surrounding room as well as the experimenter, the mazes were surrounded by a white curtain. The mazes were cleaned with 0.5% acetic acid between trials. Videos were analyzed to measure the number of arm entries and to calculate the percentage of spontaneous alternations, calculated by dividing the number of 3-arm alternations by the number of possible 3-arm alternations and multiplying the value by 100. The criteria for an arm entry was when all four limbs were within the arm. 

*Novel object recognition.* In this test, the mice were put in the open field containing two identical objects (orange wooden blocks in the shape of hexagonal prisms) for a 15 min trial. The objects were placed 10 cm apart and 15 cm from the adjacent walls of the arena. The following day, one object was replaced with a novel one (a green wooden block in the shape of a triangular prism). Mice were again allowed to explore for 15 min. Objects were affixed to the floor of the arena using masking tape. The arenas and objects were cleaned with 0.5% acetic acid between trials. Physical interaction with the object in the form of sniffing within a 2 cm proximity was coded as object exploration by hand scoring videos acquired with Noldus Ethovision software (version 17, Wageningen, The Netherlands). In addition to the percentage of time spent exploring the novel and familiar objects, a discrimination index was calculated. The time spent exploring the familiar object was subtracted from the time exploring the novel object, and the resulting number was divided by the total time spent exploring both objects. 

*Elevated zero maze.* Measures of anxiety were assessed in the elevated zero maze as described in [32]. The elevated zero maze consists of two open and two closed areas (each 35.5 cm in length; Hamilton-Kinder, Poway, CA, USA). The closed areas were surrounded by opaque walls (15 cm tall). Mice were placed into the maze in one of the open areas and allowed to explore for a single 10 min trial. The time spent in the open areas was analyzed with Noldus 17 Ethovision video tracking software.

*Light–dark test.* In the light–dark test, mice were placed in an open-field enclosure (40.64 × 40.64 cm) containing black plastic inserts covering the sides and the top 50% of the open field (Kinder Scientific, Poway, CA, USA), as described in [33]. A single opening in the wall of the insert adjacent to the open area allowed the mice to enter or exit the more anxiety-provoking light area of the maze. Active times, distance moved, and rest time were recorded for a single 10 min session. Breaks in the photo beams were used to calculate path length, active times, and rest time in the open and closed compartments of the enclosure. Mice with increased levels of anxiety in this enclosure spend less time in the lighted compartment of the maze.

*Spatial Y maze.* The spatial Y maze test had raised sides and was made of non-reflective opaque gray plastic (30 cm × 6 cm × 15 cm) (Harvard Apparatus, Panlab, Holliston, MA, USA), as described in [34]. On day 1, one arm was blocked off and mice were allowed to explore the maze for 15 min. Extra-maze spatial cues were taped on all three walls of the biosafety cabinet in which the mice were being tested. On day 2, all of the arms were accessible, and mice were allowed to explore for a 5 min trial. Videos of day 2 were analyzed to measure the number of entries and the percentage of time spent in the novel arm (the arm that was blocked off during day 1). The criteria for an arm entry was when all four limbs were within the arm.

*Fear learning and cued fear memory*. Fear conditioning was assessed over the course of 2 consecutive days using a Med Associates mouse fear conditioning system (PMED-VFC-NIR-M, Med Associates, St. Albans, Vermont) and Med Associates VideoFreeze automated scoring system, as described in [35]. Mice were placed inside the fear conditioning chamber, where chamber lights (100 lux) were turned on at the beginning of the trial. Following a 90 s habituation period, there was a 30 s (2800 Hz, 80 dB) tone (cue). A 2 s 0.35 mA foot shock was administered at 28 s, co-terminating with the tone at 30 s. After a 90 s inter-stimulus interval, there was another tone-shock pairing. On day 2, cued fear memory was assessed by placing mice in a new environment (scented with vanilla extract, cleaned with 10% isopropanol instead of 0.5% glacial acetic acid, novel floor texture covering the shock grid, and rounded walls). The mice were habituated to the new environment for 90 s (pre-tone), and exposed to the tone (cue) for 180 s.

*Grip Strength.* Grip strength was assessed using a Harvard Apparatus (Holliston, MA, USA) grip strength meter for mice. The grip strength meter allows the study of neuromuscular functions in rodents by determining the maximum force displayed by an animal. The grip strength meter was positioned horizontally and the mice were held by the tail and lowered toward the apparatus. The mice were allowed to grasp the metal grid and were then pulled backward in the horizontal plane. The force applied to the grid just before the mouse lost grip was recorded as the peak tension. We performed 3 consecutive measurements at one-minute intervals, as described in [36] (see Figure S4).

*Preparation of hippocampi and spleen for flow cytometry.* The mice were euthanized by cervical dislocation. Following the cervical dislocation, the brains were quickly removed and the hippocampi were dissected in RPMI medium (Gibco, ThermoFischer Scientific, Pittsburgh, PA, USA). The hippocampus of one hemibrain was collected in 300 μL of RPMI. Flow cytometry was performed using hippocampi from one hemibrain as described in [25], with the following modifications. To work out the conditions and as control, the spleen tissue of one mouse was used and collected in 600 μL of RPMI. The tissues were homogenized using a pestle and Dounce homogenizer on ice (30 strokes) and filtered using 40 μm Falcon Nylon Cell Strainer filters (Corning Incorporated, Corning, NY, USA) over 50 mL tubes. The Dounce tubes were washed with 5 × 1 mL of RPMI (10 × 1 mL RPMI for the spleen tissue) and the filtrates were transferred to 15 mL tubes and centrifuged at 400 rcf for 5 min. The supernatants were aspirated and the pellets were resuspended in 1 mL of Lysis buffer (BD Pharm Lyse, BD Biosciences, San Jose, CA, USA) and incubated for 5 min at room temperature. After adding 5 mL of FACS buffer (Hanks without calcium and magnesium, and with 2% goat serum), the tubes were centrifuged at 400 rcf for 5 min. The supernatants were aspirated, washed with 5 mL PBS (with calcium and magnesium), and centrifuged at 400 rcf for 3 min. The supernatants were aspirated and the pellets were incubated in the dark with ghost dye (anti-human/mouse CD11b-APC-Cy7 (Tonbo, San Diego, CA, USA), diluted 1:1000) for 30 min on ice. After adding 5 mL of FACS buffer, the tubes were centrifuged at 400 rcf for 3 min. The supernatants were aspirated and the pellets were resuspended in 100 μL of blocking antibody (anti-mouse CD16/32, diluted 1:100 in FACS buffer, eBioscience, Pittsburgh, PA, USA) and incubated on ice for 20 min. After adding 5 mL of FACS buffer, the tubes were centrifuged at 400 rcf for 3 min. The supernatants were aspirated and the pellets resuspended in 100 μL of neutrophil panel (CD11b-FITC, 1:100, eBioscience, Pittsburgh, PA, USA; Ly6G-BV570 (Biolegend, San Diego, CA, USA), 1:100; Ly6G-PerCP-Cy5.5, 1:100 (Biolegend); CD45-Violet Fluor 450, 1:100 (Tonbo, San Diego, CA, USA); and CD206-MMR-Alexa Fluor 647, 1:100 (Biolegend) and incubated on ice for 30 min. After adding 5 mL of FACS buffer, the tubes were centrifuged at 400 rcf for 3 min. The supernatants were aspirated and the pellets were resuspended in 500 μL of FACS buffer.

*Flow cytometry.* Spectral compensation was achieved by using compensation beads (ABC Anti-Mouse Bead Kit, Invitrogen, Life Technologies Corporation, Eugene, OR, USA) conjugated to the above-mentioned antibodies in 100 μL of FACS buffer and following a vendor-recommended protocol. Following vortexing the comp beads, one drop of each bottle of com beads was added and the tubes were incubated for 20 min at room temperature. After adding 3 mL of FACS buffer, the tubes were centrifugated at 400 rcf for 5 min. The supernatants were aspirated and the pellets were resuspended in 500 μL of FACS buffer.

Data for flow cytometry were acquired on a BD FAC Symphony A5 (BD Immunocytometry Systems) equipped with 405 nm (CD45, BF450; Ly6G, BV570), 488 nm (CD11b, FITC; Ly6G, PerCP), and 628 nm (CD206, AF647) excitation lasers. Data were collected and analyzed using BD FACS Diva Software v8.0.2 (BD Biosciences) and FlowJo software v10.1 (FLOWJO, Ashland, OR, USA). As female and male samples were analyzed on separate days, the percentage of the subpopulation of CD45+ Ly6G+ cells was normalized to the sex-matched sham-irradiated samples analyzed on the same day. 

Cells were first gated on FSC and SSC in order to eliminate debris. They were then gated on FSC-W and SSC-W in order to reduce the analysis of doublets. Cells that were negative for the viability marker, Ghost Red, were gated on. After this, CD45 cells were gated on and Ly5G, CD11b, and CD206 were all analyzed as a subset of CD45.

*Data analyses.* Data are expressed as mean ± SEM. Graphs were generated using GraphPad software v.8.2.0 (La Jolla, CA, USA). Data were analyzed using GraphPad and SPSS software (Version 25, Armonk, NY, USA: IBM Corp.). The data were analyzed using ANOVAs with exposure conditions as between-group factors, followed up by post hoc tests when appropriate. Performance over multiple trials was analyzed by repeated-measures ANOVA. For assessing relationships between flow cytometry and behavioral measures, Spearman correlational analyses were used.

## Figures and Tables

**Table 1:** All UHDR irradiation details ^1^.

Pulses Delivered	Average Dose Delivered	Standard Deviation
15–16	17.3 Gy	0.55 Gy
15–16	16.8	0.13 Gy
	17.7	0.21 Gy

^1^ UHDR irradiation details: 15 pulses were prescribed to reach the 18 Gy target. The overall average delivered dose was 17.3 ± 0.55 Gy. This irradiation platform occasionally delivered an additional pulse, leading to a bimodal distribution that was not biased to any cohort. Mice irradiated with 15 pulses received 16.8 Gy, and mice irradiated with 16 pulses received 17.7 Gy.

## Data Availability

Data are available upon request.

## Supplementary Materials

The following supporting information can be downloaded: https://www.mdpi.com/article/10.3390/ijms241512497/s1.

## Abbreviations

AD	Alzheimer’s Disease
ANOVA	Analysis of Variance
BV570	Brilliant™ Violet 570
CCR2	C-C chemokine receptor type 2
Cy7	Cyanine 7
FACS	Fluorescence Activated Cell Sorting
FITC	Fluorescein Isothiocyanate
GI	Gastrointestinal
Gy	Gray
Hz	Hertz
ISI	Inter-Stimulus Interval
IR	Irradiation
Ly6G	Lymphocyte antigen 6 family member G
MeV	Megaelectron Volt
m	Milli
c	Centi
μ	Micro
NOR	Novel Object Recognition
OHSU	Oregon Health & Science University
PerCP	Peridinin-Chlorophyll-protein
PBS	Phosphate-Buffered Saline
Rcf	Relative Centrifugal Force
RPMI	Roswell Park Memorial Institute
S	Seconds
SEM	Standard Error of the Mean
SPSS	Statistical Package for the Social Sciences
UHDR	Ultra-High Dose Rate
WBI	Whole Brain Irradiation
