# Supplementary material for: Effects of UHDR and Conventional Irradiation on Behavioral and Cognitive Performance and the Percentage of Ly6G+ CD45+ Cells in the Hippocampus
Source: Int J Mol Sci. 2023 Aug 6;24(15):12497. doi: 10.3390/ijms241512497 (PMC10419899; doi:10.3390/ijms241512497)
Supplement: Supplementary file 1 [file ijms-24-12497-s001.zip › ijms-2518239-supplementary.pdf]

Supplementary Material:

Effects of UHDR and Conventional Irradiation on Behavioral and Cognitive Performance and the Percentage of Ly6G+ CD45+ Cells in the Hippocampus

Ariel Chaklai, Pamela Canaday, Abigail O'Neil, Francis A. Cucinotta, Austin Sloop, David Gladstone, Brian Pogue, Rongxiao Zhang, Jacob Sunnerberg, Alireza Kheirolla, Charles R. Thomas, Jr., P. Jack Hoopes, and Jacob Raber

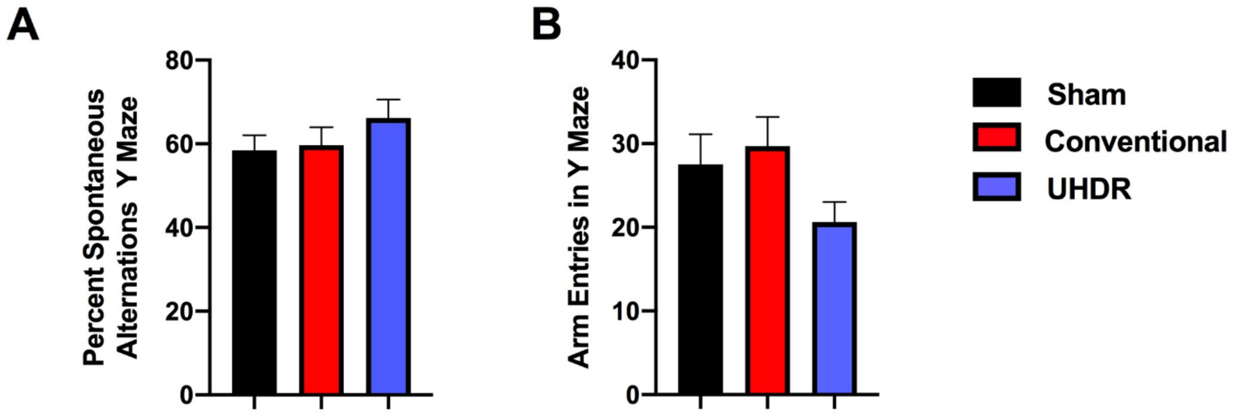

Figure S1. There was no effect of radiation on spontaneous alternation (**A**) or entries (**B**) (F = 1.893, p = 0.2128) in the Y maze.

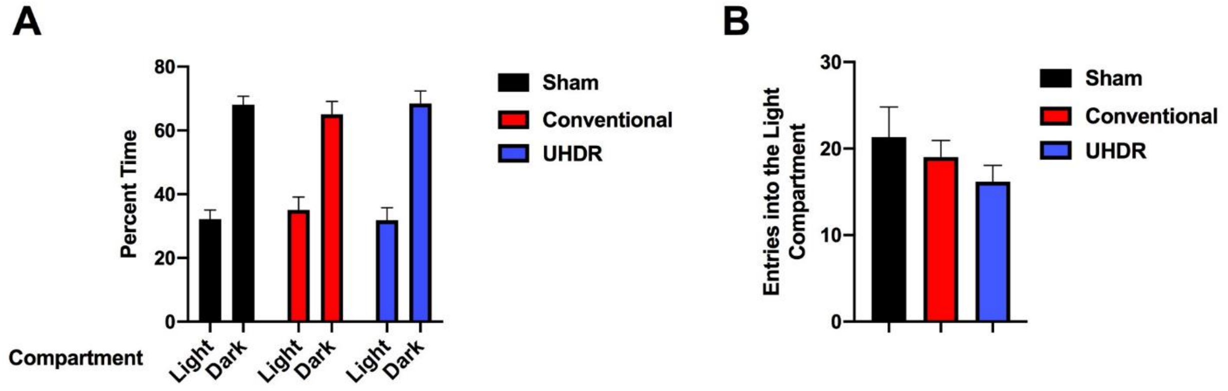

Figure S2. In the light-dark test, there was no effect of radiation on the percent time spent in the two compartments (**A**) or the number of entries into the light compartment (**B**).

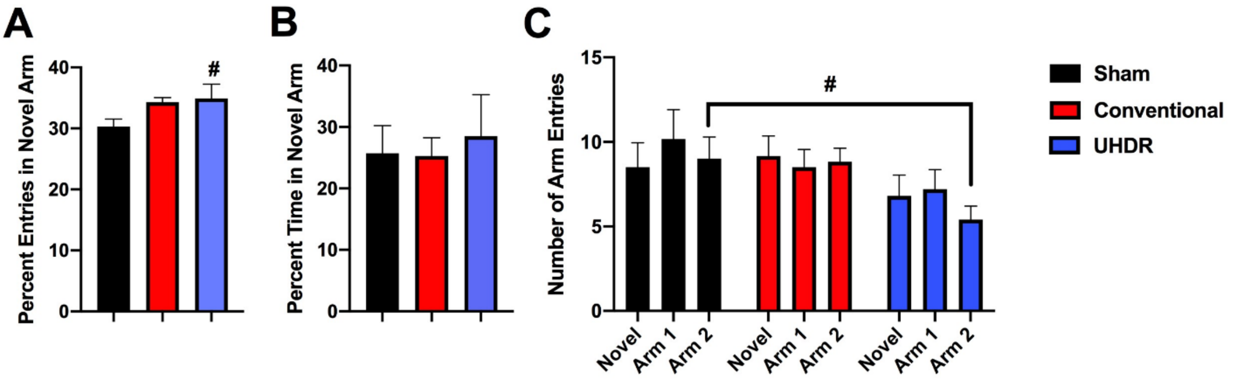

Figure S3. A. In the spatial Y maze, there was a trend towards a higher percent entries in UHDR- than sham-irradiated mice. # p =0.0830). **B**. There was no effect of radiation on the percent time spent in the novel arm. **C**. For the number of arm entries, there was a radiation × arm interaction ($F(4, 28) = 2.812$, $p = 0.0443$, with a trend towards less arm 2 entries in UHDR- than sham-irradiated mice. # $p = 0.08$, Dunnett's).

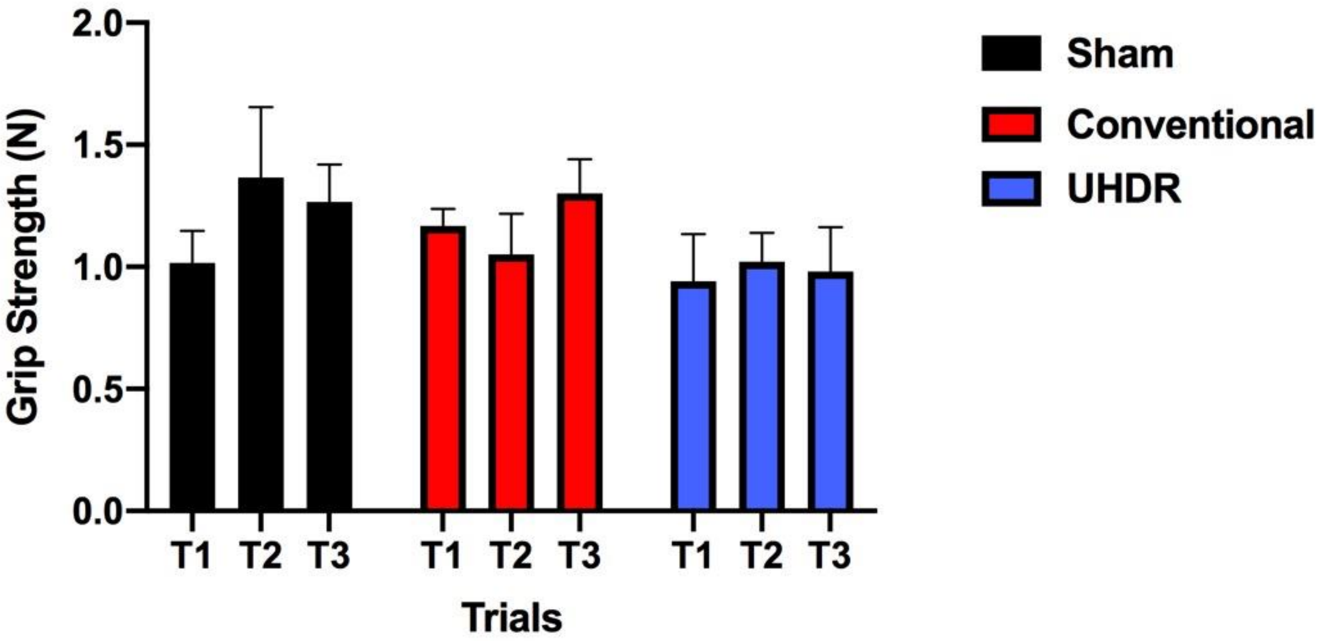

Figure S4. There was no effect of radiation on relative grip strength.
